# Mapping spatial and social inequities of long COVID across the United States: a retrospective cohort study

**DOI:** 10.1016/j.lana.2026.101401

**Published:** 2026-02-13

**Authors:** Zhetao Chen, Bingnan Li, Yewen Chen, Jialing Liu, Fangzhi Luo, Kehinde Olawale Ogunyemi, Yang Ge, Yuan Ke, Yang Yang, Xianyan Chen, Ye Shen, the National COVID Cohort Collaborative, Adam B. Wilcox, Adam M. Lee, Alexis Graves, Alfred (Jerrod) Anzalone, Amin Manna, Amit Saha, Amy Olex, Andrea Zhouss, Andrew E. Williams, Andrew M. Southerland, Andrew T. Girvin, Anita Walden, Anjali Sharathkumar, Benjamin Amor, Benjamin Bates, Brian Hendricks, Brijesh Patel, G. Caleb Alexander, Carolyn T. Bramante, Cavin Ward-Caviness, Charisse Madlock-Brown, Christine Suver, Christopher G. Chute, Christopher Dillon, Chunlei Wu, Clare Schmitt, Cliff Takemoto, Dan Housman, Davera Gabriel, David A. Eichmann, Diego Mazzotti, Donald E. Brown, Eilis Boudreau, Elaine L. Hill, Emily Carlson Marti, Emily R. Pfaff, Evan French, Farrukh M. Koraishy, Federico Mariona, Fred Prior, George Sokos, Greg Martin, Harold P. Lehmann, Heidi Spratt, Hemalkumar B. Mehta, J.W. Awori Hayanga, Jami Pincavitch, Jaylyn Clark, Jeremy Richard Harper, Jessica Yasmine Islam, Jin Ge, Joel Gagnier, Johanna J. Loomba, John B. Buse, Jomol Mathew, Joni L. Rutter, Julie A. McMurry, Justin Guinney, Justin Starren, Karen Crowley, Katie Rebecca Bradwell, Kellie M. Walters, Ken Wilkins, Kenneth R. Gersing, Kenrick Cato, Kimberly Murray, Kristin Kostka, Lavance Northington, Lee Pyles, Lesley Cottrell, Lili M. Portilla, Mariam Deacy, Mark M. Bissell, Marshall Clark, Mary Emmett, Matvey B. Palchuk, Melissa A. Haendel, Meredith Adams, Meredith Temple-O’Connor, Michael G. Kurilla, Michele Morris, Nasia Safdar, Nicole Garbarini, Noha Sharafeldin, Ofer Sadan, Patricia A. Francis, Penny Wung Burgoon, Philip R.O. Payne, Randeep Jawa, Rebecca Erwin-Cohen, Rena C. Patel, Richard A. Moffitt, Richard L. Zhu, Rishikesan Kamaleswaran, Robert Hurley, Robert T. Miller, Saiju Pyarajan, Sam G. Michael, Samuel Bozzette, Sandeep K. Mallipattu, Satyanarayana Vedula, Scott Chapman, Shawn T. O'Neil, Soko Setoguchi, Stephanie S. Hong, Steven G. Johnson, Tellen D. Bennett, Tiffany J. Callahan, Umit Topaloglu, Valery Gordon, Vignesh Subbian, Warren A. Kibbe, Wenndy Hernandez, Will Beasley, Will Cooper, William Hillegass, Xiaohan Tanner Zhang

**Affiliations:** a Department of Epidemiology & Biostatistics, College of Public Health, University of Georgia, Athens, GA, USA; b Department of Statistics, Franklin College of Arts and Sciences, University of Georgia, Athens, GA, USA

**Keywords:** Public health, Long COVID, Spatiotemporal disparities, Spatial and social inequities

## Abstract

**Background:**

Long COVID affects a substantial portion of the U.S. population. The emergence of the Omicron variant and persistent sociodemographic disparities may contribute to temporal and regional variation in long COVID risk. However, such spatiotemporal variation and related social determinants remain poorly characterized. This study aimed to examine spatiotemporal patterns of county-level long COVID incidence and to identify sociodemographic factors associated with these patterns before and after the emergence of the Omicron variant.

**Methods:**

This retrospective study utilized data from the National COVID Cohort Collaborative (N3C), covering 5,652,474 COVID-19 cases from 2020 to 2024 and 41,694 long COVID cases across 1063 U.S. counties from 2021 to 2024. Temporal patterns of long COVID were analyzed before and after the Omicron variant's emergence, and spatial patterns were assessed using Moran's I and Getis statistics. Bayesian spatial random effect models were employed to evaluate the associations between long COVID incidence and sociodemographic factors such as economic vulnerability, healthcare access, and mobility.

**Findings:**

Among 4,070,879 COVID-19 cases analyzed, quarterly long COVID incidence ranged from 0.015% to 14.29%. Before the emergence of the Omicron variant, incidence was 204 cases per 10,000 COVID-19 cases, compared with 248 cases per 10,000 COVID-19 cases after Omicron emergence (p < 0.001). Based on the Local Moran's I statistic, 48.8% (328 of 673) of counties showed significant spatial correlation (p < 0.05) after Omicron's emergence, up from 43.5% (293 of 673) prior. High-risk areas became more concentrated in inland regions, while low-risk areas clustered along the East Coast. Long COVID incidence was significantly associated with economic vulnerability, limited healthcare access, and mobility constraints, with these sociodemographic disparities consistently driving its spatial disparities over time. Subregional analyses revealed distinct regional differences in social drivers.

**Interpretation:**

These findings highlight pronounced spatiotemporal and regional disparities in long COVID incidence across the United States. Targeted public health interventions, particularly in economically and geographically vulnerable regions, are essential to ensure equitable access to diagnosis, care, and resource allocation.

**Funding:**

National Center for Advancing Translational Sciences; National Institutes of Health; National Science Foundation.

**Research in context**

**Evidence before this study**

Long COVID, or post-acute sequelae of SARS-CoV-2 infection (PASC), has emerged as a persistent public health concern with multisystem effects and substantial population burden. We searched PubMed from 2020 to 2025 using the terms (“long COVID” OR “post-COVID-19 condition” OR “PASC” OR “post-acute sequelae”) combined with “risk”, “spatial”, and “social”. Most previous studies have focused on individual-level demographic and clinical risk factors, providing limited understanding of the geographic and social determinants of long COVID incidence. Although national surveys and regional analyses have suggested spatial and socioeconomic disparities across the United States, these studies often relied on self-reported data or restricted geographic coverage. Few investigations have examined how county level social vulnerability interacts with virological transitions, such as the emergence of the Omicron variant, to influence regional variations in long COVID incidence.

**Added value of this study**

Using harmonized electronic health record data from over 5.6 million adults with confirmed COVID-19 across the National COVID Cohort Collaborative (N3C), this study provides the first nationwide, county-level assessment of the spatiotemporal and social determinants of long COVID incidence in the United States. By integrating county level social vulnerability indicators with longitudinal clinical data, we identified key demographic and structural factors, such as rurality, poverty levels, disability prevalence, institutional living environments, minoritised population composition, vaccination coverage, and residential crowding, that consistently influenced long COVID risk. Moreover, by comparing pre-Omicron and Omicron periods, our findings reveal how the relative importance of these factors evolved with changing viral characteristics and population immunity.

**Implications of all the available evidence**

Our findings highlight that long COVID is not only a biomedical condition but also a manifestation of persistent structural inequities in healthcare access and social determinants of health. Addressing these disparities requires policies that ensure equitable diagnosis, management, and rehabilitation across regions. Areas with a high burden of long COVID should receive greater investment in specialized clinics and chronic care programs, while under-diagnosed regions need improved surveillance and clinician training. By embedding equity into long COVID policy and healthcare delivery, public health systems can reduce regional disparities, improve patient outcomes, and strengthen resilience against future post-viral conditions.

## Introduction

Long COVID, or post-acute sequelae of SARS-CoV-2 infection (PASC), has become a major public health concern due to its long-term impacts.^1^^,^^2^ In 2022, approximately 6.9% of adults reported having ever experienced long COVID, and 3.4% were currently experiencing it, illustrating the substantial prevalence and persistence of this condition.^3^ These long-term symptoms can involve multiple physiological systems, such as respiratory, neurological, cardiovascular, and gastrointestinal, leading to fatigue, cognitive impairment, shortness of breath, and other chronic issues.^4^ Although many epidemiological studies have explored individual-level risk factors for long COVID-19,^5^^,^^6^ our understanding of its spatial and temporal incidence dynamics remains limited.

The distribution of long COVID exhibits complex spatial and temporal disparities. The spread of acute COVID-19 was highly uneven across regions,^7^ and emerging evidence suggests that long COVID prevalence also varies geographically.^8^^,^^9^ According to a June 2022 survey conducted by the Centers for Disease Control and Prevention (CDC), the prevalence of current long COVID symptoms among the United States (U.S.) adults varied substantially across states, ranging from 4.5% in Hawaii to 12.7% in Kentucky.^10^ Additionally, virological transitions have fundamentally altered COVID-19 dynamics.^11^ The Omicron variant rapidly became dominant in early 2022 and introduced major shifts in transmissibility, infection severity, population immunity levels, and clinical manifestations. Recent clinical studies have shown that the risk and symptom profile of long COVID differ significantly between pre-Omicron and Omicron infections, with markedly lower prevalence observed among Omicron cases.^12^, ^13^, ^14^ Beyond these patterns, socioeconomic and demographic factors can play a crucial role in accounting for long COVID risk.^10^^,^^15^ Prior studies have shown that prevalence differs across age groups and racial/ethnic populations.^16^ However, previous studies have rarely examined the spatiotemporal patterns of long COVID incidence and how these factors dynamically influence spatial disparities in long COVID incidence.

In this study, we analyzed county-level long COVID data from the National COVID Cohort Collaborative (N3C) and sociodemographic data from the Social Vulnerability Index (SVI), covering 1063 U.S. counties over the period 2021–2024. Our analysis advances prior work by providing a detailed spatiotemporal assessment and evaluating the influence of community-level social factors on incidence patterns. These findings can help guide targeted public health strategies and inform equitable healthcare resource allocation.

## Methods

### Study population and data collection

We constructed two datasets from the N3C database (version 185), one for acute COVID-19 and one for long COVID, based on daily EHR data from July 1, 2020 to March 31, 2024 (Supplementary Figure S1). The data included individual-level records of confirmed cases, ZIP codes of site locations, and associated demographics, contributed by 62 sites across U.S. counties. Long COVID cases were identified using the International Classification of Diseases-10-Clinical Modification (ICD-10-CM)^17^ code B94.8 before October 1, 2021 and code U09.9 thereafter to account for changes in clinical coding practices over time.^18^ We defined the population at risk as individuals with a recorded acute COVID-19 diagnosis (ICD-10-CM code U07.1).

Due to the sparse spatial distribution of reported long COVID cases across the U.S (Supplementary Figure S4), we conducted analyses at the county level rather than the ZIP code level to ensure robust and stable incidence estimates. To reduce bias and variance in assessing long COVID incidence, we employed two complementary strategies to restrict the study population. First, we included only counties with at least one reported long COVID case between 2020 and 2024 to mitigate potential detection bias across regions. In the second strategy, we further restricted our analysis to counties with ≥20 reported acute COVID-19 cases to avoid inflated incidence estimates, as counties with very few cases may produce incidences approaching or even equal to 1, which cannot reflect the true situation. This threshold was selected following guidance from the U.S. National Center for Health Statistics.^19^ Moreover, to enable valid pre-post comparisons around the Omicron-dominant period, we further restricted the related comparison analysis to 673 counties with available long COVID data both before and after this period.

To understand factors potentially influencing long COVID incidence, we incorporated socioeconomic and demographic variables from SVI, developed by the Centers for Disease Control and Prevention and the Agency for Toxic Substances and Disease Registry.^20^ We used two versions of the SVI data to align with our pre- and post-Omicron analyses: the 2022 SVI, based on the 5-year (2018–2022) American Community Survey (ACS), was used for the post-Omicron period, and the 2020 SVI, based on the 5-year (2016–2020) ACS, was used for the pre-Omicron period. This approach ensures that the socioeconomic context used in each analysis reflects the corresponding time frame as accurately as possible, which is particularly important given potential shifts in community characteristics over the course of the pandemic. SVI was designed to identify communities that may need support before, during, or after public health emergencies. Besides, we incorporated the Rural-Urban Continuum Codes to reflect geographic accessibility and differences in healthcare resource distribution across counties.^21^ Finally, we included the first dose COVID-19 vaccination rate as a proxy of county-level immunity and healthcare engagement. Overall, all 16 SVI-related variables, along with Rural-Urban Continuum Codes and first dose COVID-19 vaccination rate were analyzed in this study. These 18 variables are summarized to Supplementary Table S1. In particular, the SVI variables reflected socioeconomic status, household characteristics, racially and ethnically minoritised status, housing type, and access to transportation.^22^

### Study outcomes

The incidence of long COVID was used as the outcome of interest. A minimum 28-day separation between acute COVID-19 and long COVID diagnosis was required, consistent with CDC guidance and prior studies indicating this temporal threshold improves diagnostic specificity.^23^^,^^24^ Individuals were considered at risk for up to 180 days after acute infection, based on the observation that >77% of long COVID diagnoses occur within this time window in N3C data (Supplementary Figure S3). To assess evolution of long COVID incidences over time, we employed a dynamic incidence risk calculation method (Supplementary Figure S2). By comparing long COVID EHR cases on specific time periods across various spatial regions with the corresponding at-risk acute COVID-19 populations, we derived two outcomes for the long COVID incidence. One was the incidence before and after the Omicron-dominant period (i.e., January 2022), which was set based on the standard provided by the WHO.^25^ The other outcome was the quarterly incidence computed using three-month intervals based on natural calendar months given emerging evidence on the seasonal variability of COVID-19.^26^

### Statistical analysis

To assess spatial local patterns in long COVID incidence across U.S. counties, we employed both local Moran's I and Getis statistics^27^^,^^28^ (Supplementary Figure S5), with a statistical significance level of 0.05. Local Moran's I quantifies the degree of similarity or dissimilarity between a county and its neighbors, with positive values indicating similarity and negative values indicating dissimilarity. In contrast, Getis identifies statistically significant spatial clusters of high (hot spots) or low (cold spots) incidence.

For the counties that met the complementary strategies described earlier, there were no missing data in either the long COVID incidence outcomes or the associated covariates. All subsequent modeling was conducted on the complete dataset. To accurately estimate potential relationships between variables and outcomes, simple stepwise regression methods were used to identify a subset of variables that best explained the variation in long COVID incidence. Given the right-skewed distribution of long COVID incidence (Supplementary Figure S9), we applied a logarithmic transformation to stabilize variance and improve model performance. We further developed spatial random effects models to account for regional heterogeneity, which can be written as:

$$y_{i}=X_{i}\beta \;+\;W_{i\in S_{g}}\;+\;\epsilon_{i}\text{,}$$

where $y_{i}$ is the incidence of the i-th county, X is the covariables selected via stepwise regression models. The coefficients derived from this model were also compared with those from the full model that includes all 18 covariates. To avoid confounding introduced by spatially correlated random effects, we modeled the zero-mean Gaussian spatial random effect $W_{i\in S_{g}}$ as subregion-level independent errors, with variances $\tau_{g}^{2}$ varying across subregions for g=1,⋯,5 (Supplementary Figure S6). The term $\epsilon_{i}$ represents zero-mean Gaussian errors with the variance of $\tau^{2}$. To avoid additional potential multicollinearity among covariables, under a Bayesian setting, we used a ridge-like prior for the coefficients β to stabilize estimates, specified as zero-mean Gaussian with a small variance of 0.1.^29^ All analyses were conducted using R statistical software (version 4.4.0).^30^

### Ethics approval

The IRB ID of this project is PROJECT00007079, and the Data Use Request (DUR) ID is DUR-6E0E8C9. The N3C Data Enclave is managed under the authority of the NIH; information can be found at https://ncats.nih.gov/n3c/resources.

### Role of the funding source

The funders had no role in the study design, data collection and analysis, decision to publish, or preparation of the manuscript.

## Results

### Overall description

**Fig. 1:**
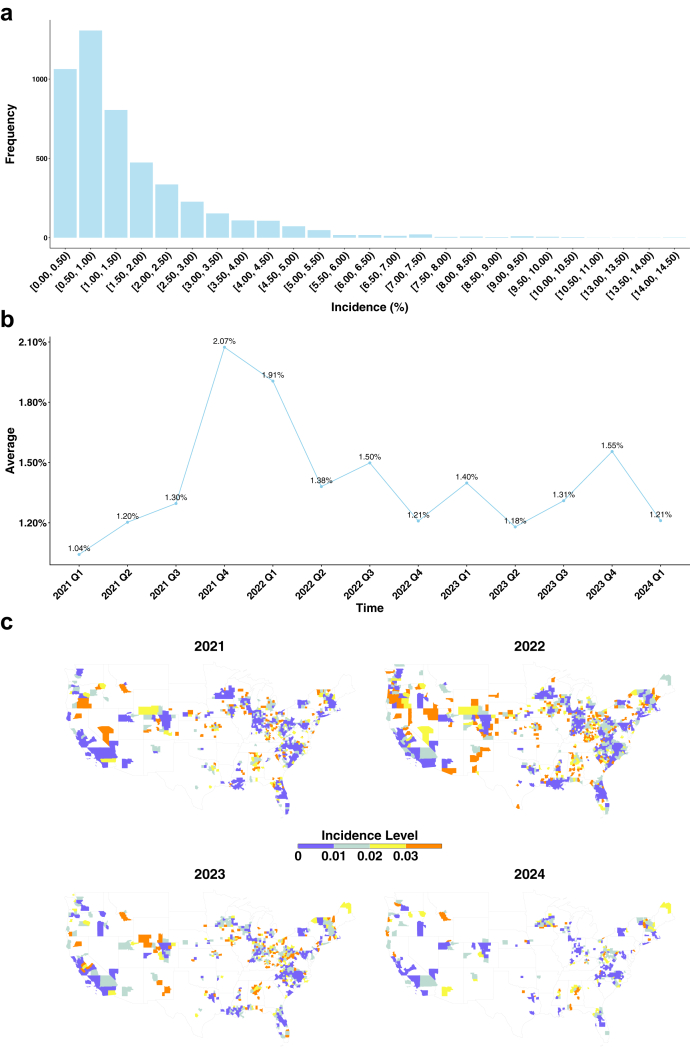
Long COVID incidences at county-level. (a) Distribution across incidence intervals; (b) Time-trend of average incidence across counties from January 2021 to March 2024; (c) County-level incidence maps (2021–2024).

From 2020 to 2024, a total of 5,652,474 COVID-19 cases were recorded in the N3C database, among which 41,694 long COVID cases were identified from 2021 to 2024 based on the dynamic incidence risk described in Supplementary Figure S2, resulting in an overall incidence risk of 0.74% across the study period. The quarterly incidence of long COVID ranged from 0.015% to 14.29%, with 66.0% of counties recording incidence rates in the interval [0.015%, 1.50%] (Fig. 1a). The average incidence across counties peaked in the fourth quarter of 2021 (October–December) at 2.07%, followed by another high in the first quarter of 2022 (January–March) at 1.91%, before declining thereafter (Fig. 1b). These two peaks coincided with the period when the Omicron variant rapidly replaced Delta as the dominant strain in the U.S. during 2022 Q1. A Wilcoxon signed-rank test (p < 0.0001) indicated a statistically significant difference in long COVID incidence before and after the Omicron-dominant period, suggesting a shift in the trend associated with the emergence of Omicron. Spatially, the distribution of long COVID incidence exhibited clear temporal variation from 2021 to 2024, with higher-incidence areas initially concentrated in the West and parts of the Northeast, gradually expanding into the Midwest and South during 2022–2023, and becoming more spatially fragmented by 2024 (Fig. 1c).

To further examine these spatial dynamics at a regional scale, we adopted a commonly used five-region classification of the U.S.^31^(Supplementary Figure S6). Most counties across five U.S. regions exhibited a substantial increase in long COVID incidence following the Omicron surge (Supplementary Table S2). Specifically, more than two-thirds of counties in each region experienced an increase with the Northeast showing the highest proportion (75%) and the South showing the lowest (66.67%). Despite regional variation, the overall proportion of counties with increased incidence was moderately high at 68.95%, highlighting the significant epidemiologic shift associated with the emergence of Omicron. In detail, the patterns of increasing or decreasing county-level incidence before and after the Omicron-dominant period exhibited local spatial similarity (Supplementary Figure S7). Moreover, the incidence varied notably across regions (Supplementary Table S3). The West consistently exhibited the highest average incidence, increasing from 2.42% before Omicron to 2.86% after its emergence. The Midwest and Northeast also experienced considerable increases, with post-Omicron incidences reaching 2.79% and 2.45%, respectively. Interestingly, while the South maintained a moderate level of incidence, increasing from 2.21% to 2.53%, the Southwest was the only region where the average incidence slightly declined after the Omicron surge, from 1.85% to 1.78%.

### Spatial correlation analysis

Local Moran's I analysis revealed that the total proportion of counties with significant correlations increased from 43.5% before the emergence of Omicron to 48.8% afterward (Supplementary Table S4). Positive spatial autocorrelation was more prevalent than negative in both periods. Specifically, the proportion of counties showing positive correlations rose from 32.7% to 35.4%, while the proportion with negative correlations increased from 10.8% to 13.4%. Regionally, the proportion of counties with positive correlations decreased in the Northeast (from 43.3% to 21.7%) and South (from 51.6% to 50.8%), remained relatively stable in the Midwest (from 22.8% to 23.2%), and increased notably in the West (from 11.1% to 32.2%) and Southwest (from 0.0% to 38.2%). For negative correlations, the proportion declined slightly in the South (from 14.7% to 14.3%), Northeast (from 8.3% to 5.0%), and Southwest (from 11.8% to 8.8%), but increased in the Midwest (from 7.2% to 12.7%) and West (from 11.1% to 20.0%). Overall, counties with significant spatial correlations were primarily located in the Middle and eastern regions (Supplementary Figure S8).

### High- and low-risk areas

**Fig. 2:**
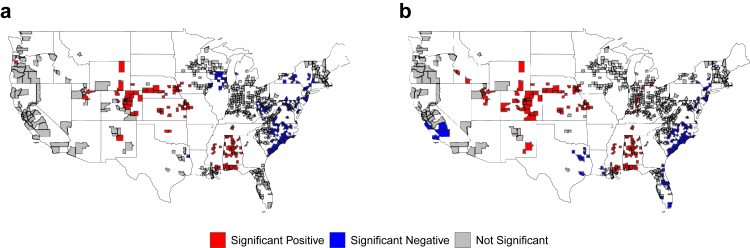
Spatial clustering of long COVID incidence across U.S. counties based on Getis statistic before (a) and after (b) Omicron dominance, at a statistical significance level of 0.05.

Getis statistics identified notable shifts in the spatial distribution of both high-risk and low-risk areas for long COVID incidence before and after the dominance of the Omicron variant (Table 1). The total proportion of counties classified as high-risk increased markedly from 14.9% to 27.2%. Regionally, the Midwest experienced the largest increase in high-risk counties, rising from 16.9% to 35.9%, followed by the West (22.2%–40.0%) and the South (14.7%–23.8%). In contrast, the Northeast had no high-risk counties identified in either period, and the Southwest showed a slight decrease from 8.8% to 5.9%. Conversely, the total proportion of counties identified as low-risk declined from 28.7% to 21.5%. The South and Northeast showed decreases in low-risk counties from 51.6%–41.3% to 51.7%–26.7%, respectively. The Midwest saw a complete drop from 13.1% to 0.0%, while the West increased slightly from 0.0% to 12.2%. Notably, the Southwest showed a substantial rise in low-risk counties, from 2.9% to 41.2%. Overall, the results indicate an expansion of high-risk clusters, especially across the Midwest and West, after the emergence of the Omicron variant, whereas low-risk areas became more concentrated in the Southwest, as visualized in Fig. 2.

**Table 1:** Changes in the number and proportion of counties with high and low long COVID incidence risk before and after Omicron dominance across different subregions of the United States (673 counties).

Region	The number (proportion^a^) of counties in high risk		The number (proportion) of counties in low risk	
	Before	After	Before	After
South	37 (14.7%)	60 (23.8%)	130 (51.6%)	104 (41.3%)
Northeast	0 (0.0%)	0 (0.0%)	31 (51.7%)	16 (26.7%)
Midwest	40 (16.9%)	85 (35.9%)	31 (13.1%)	0 (0.0%)
West	20 (22.2%)	36 (40.0%)	0 (0.0%)	11 (12.2%)
Southwest	3 (8.8%)	2 (5.9%)	1 (2.9%)	14 (41.2%)
Total	100 (14.9%)	183 (27.2%)	193 (28.7%)	145 (21.5%)

a Proportion = (Number of high- or low-risk counties)/(Total number of counties in the region).

### Associations between social vulnerability factors and long COVID incidence

**Fig. 3:**
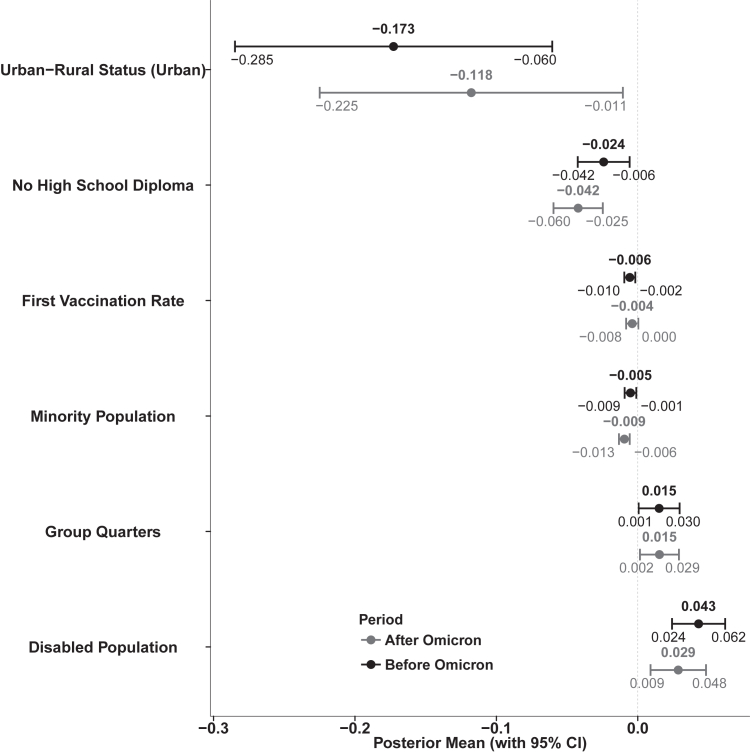
Posterior estimates with 95% credible intervals (CIs) for regression coefficients that measure the relationships between social vulnerability factors and long COVID incidence based on Bayesian spatial random effect models.

Many variables exhibited high correlations, with Pearson coefficients exceeding 0.65 (Supplementary Figure S10). Such high correlations can lead to unstable estimation of regression relationships, including potential sign reversals and altered significance levels.^32^ We conducted a stepwise regression with the Akaike Information Criterion (AIC) to reduce estimation variance. Stepwise regression identified different sets of variables for the periods before and after Omicron dominance (Supplementary Tables S5 and S6). The spatial random effect models were then developed using the selected variables. Fig. 3 presents the results for variables that were selected in both periods, while variables that differed between the two periods are shown in Supplementary Figure S12. Urban counties consistently exhibited lower long COVID incidence than rural counties in both the pre- and post-Omicron periods (−0.173, 95% CI: −0.285 to −0.061 before; −0.118, −0.225 to −0.011 after). Counties with a higher proportion of adults without a high school diploma also showed significant negative associations across both periods (−0.024, −0.042 to −0.006 before; −0.042, −0.060 to −0.025 after), as did those with a higher proportion of racially and ethnically minoritised populations (−0.005, −0.009 to −0.001 before; −0.009, −0.013 to −0.006 after) and a higher proportion of first vaccination (−0.006, −0.010 to −0.002 before; −0.004, −0.008 to 0 after). In contrast, a higher proportion of disabled residents was significantly positively associated with long COVID incidence only before Omicron (0.043, 0.024–0.062), with a weaker but still significant association observed afterward (0.029, 0.009–0.048). Similarly, counties with more residents living in group quarters showed a significant positive association in both periods (0.015, 0.001–0.030 before; 0.015, 0.002–0.029 after). Additional variables that were significantly associated with long COVID incidence but only in one period are shown in Supplementary Figure S12. For example, the proportion of the population aged over 65 (−0.019, −0.032 to −0.006), multi-unit housing (−0.012, −0.020 to −0.003), limited English proficiency (0.045, 0.013–0.077), and the proportion below 150% of the federal poverty level (0.015, 0.004–0.026) were significant only after Omicron, whereas only housing cost burden (0.017, 0.004–0.030) were significant only before Omicron. In particular, the spatial random effect methods improved model fit and strengthened the robustness of statistical inference based on histograms of residuals (Supplementary Figure S11).

Furthermore, we compared the above results with an additional analysis using the full set of 18 covariates without any variable selection (Supplementary Figure S13). Overall, the directions of most associations were consistent with those from the stepwise-selected models. While several differences did emerge: the associations for minoritised population share and housing cost burden were no longer statistically significant in the pre-Omicron period, and the proportion of residents living in group quarters was no longer significant in either period. In contrast, crowded housing, excluded by stepwise selection, became a significant negative predictor of long COVID incidence in the post-Omicron period. To further evaluate the contribution of cumulative vulnerability, we examined the Overall Social Vulnerability Score, a composite metric summarizing the combined effects of all SVI components (Supplementary Figure S14). This measure demonstrated a significant negative association with long COVID incidence only in the post-Omicron period (−0.308, 95% CI: −0.485 to −0.131).

### Subregional stratified analysis of social vulnerability and long COVID incidence

We observed substantial regional variation in the magnitude of regression coefficients as well as in their consistency across different periods (Supplementary Figures S15 and S16). Urban residence was protective only in the West before Omicron (−0.290, 95% CI: −0.551 to −0.028), while the proportion of adults without a high school diploma showed stable negative associations in the Midwest (−0.075, −0.116 to −0.034 before; −0.059, −0.092 to −0.027 after) and Southwest (−0.094, −0.184 to −0.004 before; −0.071, −0.137 to −0.005 after). First vaccination rate also demonstrated consistent protective associations but only in the Midwest (−0.009, −0.016 to −0.001 before; −0.014, −0.025 to −0.003 after) and South (−0.007, −0.013 to −0.002 before; −0.008, −0.015 to −0.001 after). Positive associations with disabled population share appeared in multiple regions, including the Midwest (0.093, 0.052–0.134 before), South (0.030, 0.002–0.057 before), Southwest (0.115, 0.023–0.207 before; 0.093, 0.018–0.168 after), and in the Northeast after Omicron (0.121, 0.033–0.209).

Distinctive directional differences also emerged across regions. Before Omicron, minoritised population share was positively associated with long COVID in the Midwest (0.015, 0.002–0.028) and Southwest (0.024, 0.002–0.047) but negatively associated in the South (−0.007, −0.013 to −0.002) and West (−0.012, −0.024 to −0.001), highlighting region-specific social contexts. Additional one-period associations were also identified: in the South, lack of vehicle access was negatively associated with long COVID before Omicron (−0.044, −0.076 to −0.013), whereas after Omicron, higher proportions of older adults were protective in the South (−0.027, −0.045 to −0.010). Moreover, counties with more residents with limited English proficiency exhibited increased incidence in the Midwest (0.102, 0.011–0.193) and Southwest (0.113, 0.029–0.197) only after Omicron.

### Impacts of sociodemographic disparities on the long COVID incidence

**Fig. 4:**
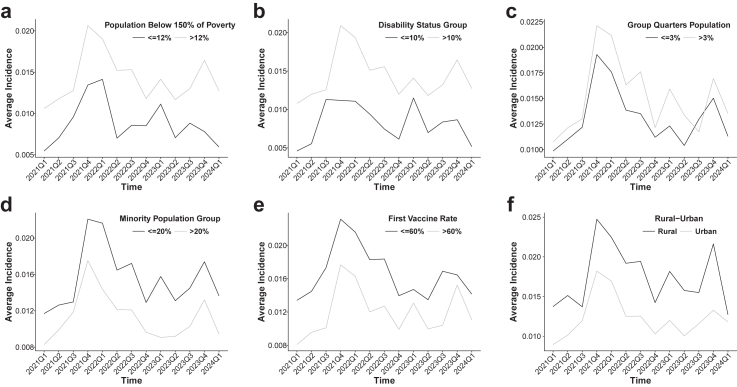
Temporal trends in long COVID incidence across different levels of six social vulnerability factors: (a) poverty status (population below 150% of the federal poverty level), (b) disability prevalence, (c) residence in group quarters, (d) racial and ethnic minority composition, (e) COVID-19 vaccination coverage, and (f) rural-urban classification.

Fig. 4a–f presents the temporal trends in long COVID incidence stratified by selected SVI-related factors, with patterns broadly consistent with the findings of spatial random effect models. Counties with a higher proportion of residents living below 150% of the poverty level (>12%) consistently exhibited higher long COVID incidence than those with lower poverty levels (≤12%) (Fig. 4a). Similarly, counties with a greater proportion of disabled residents (>10%) experienced higher incidence compared to those with lower disability prevalence (≤10%) across most quarters (Fig. 4b). In Fig. 4c, counties with more residents living in group quarters (>3%) also showed elevated incidence relative to those with ≤3%. Interestingly, Fig. 4d indicates that counties with higher minoritised population proportions (>20%) tended to have lower long COVID incidence than those with lower proportions (≤20%), particularly after 2022. Counties with higher vaccination rates (>60%) consistently reported lower incidence than those with lower rates (≤60%) (Fig. 4e). Finally, rural counties exhibited higher long COVID incidence than urban counties throughout the study period, with a marked divergence following the emergence of Omicron (Fig. 4f). In particular, the threshold for each variable was selected based on comparison analysis to ensure that, under the given threshold, the difference in long COVID incidence related to the variable was statistically significant in most of the six subregions (Supplementary Table S7). This approach ensured the robustness of the results above.

## Discussion

This study offers a comprehensive view of long COVID incidence by analyzing its spatiotemporal patterns and associations with area-level socioeconomic and demographic factors, extending beyond traditional individual-level analyses. Using a large U.S. dataset of 5,652,474 COVID-19 from 2020 to 2024 and 41,694 long COVID cases from 2021 to 2024, we identified significant spatiotemporal differences in its epidemiology. Spatial clustering of incidence was observed across many counties (p < 0.05). Notably, low-incidence areas were more concentrated on the U.S. East Coast, while high-incidence areas distributed inland regions, likely reflecting pre-existing disparities in health (healthcare and public health) services coverage and available social infrastructure. This epidemiological pattern may in part be explained by evidence of socioeconomic and demographic clustering in these regions, where Americans of higher socioeconomic status have been reported to be migrating to the coastal regions while those with lower socioeconomic status are moving to the inland regions.^33^ These findings further suggest that low-income and underserved populations in inland regions may face a disproportionately higher risk of developing long COVID. This elevated vulnerability is likely driven by the higher prevalence of contributing factors in these communities, such as comorbidities and smoking.^34^^,^^35^

This study identifies SVI factors associated with long COVID and clarifies their role in spatiotemporal disparities. Our analysis identified rurality, poverty levels, disability prevalence, institutional living environments, minoritised population composition, vaccination coverage, and residential crowding as key factors influencing long COVID incidence, with varying effects across time periods. Importantly, the spatially identified determinants were highly consistent with established individual-level risk factors for long COVID. For example, disability status, poverty, rural residence, and limited vaccination protection have been repeatedly associated with prolonged post-acute symptoms at the individual level,^11^^,^^13^^,^^14^ and our findings indicate that these individual-level risk factors are usually linked with county-level vulnerabilities, with higher long COVID incidence observed in counties where these susceptible populations are more concentrated. Specifically, we consistently observed higher long COVID incidence between 2021 and 2024 in counties characterized by rural status, higher disability prevalence, and a larger share of residents in institutional living environments. Counties with higher poverty levels and greater residential crowding became more strongly associated with higher long COVID incidence after the emergence of the Omicron variant. This finding highlights the disproportionate burden of long COVID among economically vulnerable communities. Existing healthcare and social determinants of health inequities likely played a critical role in shaping these patterns, as lower healthcare access and higher economic vulnerability contribute to prolonged post-acute symptoms and delayed medical diagnosis and treatment.^36^ In contrast, higher COVID-19 vaccination coverage was associated with lower long COVID incidence primarily in the pre-Omicron period, suggesting a potential early protective effect against long-term complications. Interestingly, counties with higher minoritised populations and lower educational attainment showed lower reported incidence. This may reflect underdiagnosis or ecological bias rather than lower individual risk, as county-level metrics may not reflect the characteristics of individuals in the N3C dataset. Moreover, individuals from minoritised backgrounds or with lower education levels may be less likely to seek care for persistent post-COVID symptoms due to health literacy challenges, language barriers, limited healthcare access, or financial and structural obstacles. As a result, these populations are more likely to be underrepresented in EHR-based long COVID case definitions. As such, future studies leveraging N3C data should interpret these associations with caution and consider the potential impact of ecological bias and data representativeness when evaluating racial and ethnic disparities or levels of educational attainment in long COVID burden. Sensitivity analyses using the full set of 18 covariates without variable selection produced broadly consistent results, confirming the robustness of our primary findings. However, several differences emerged, including the loss of statistical significance for minoritised population share and housing cost burden in the pre-Omicron period, and for group quarters across both periods. Interestingly, crowded housing, excluded by stepwise selection, became a significant negative predictor of long COVID incidence after Omicron, potentially reflecting changes in exposure patterns or healthcare-seeking behaviors in highly populated areas during the later stages of the pandemic. Furthermore, the Overall Social Vulnerability Score demonstrated a significant negative association with long COVID incidence only in the post-Omicron period. This pattern was likely related to complex interactions between weights or correlations among components of SVI. Furthermore, our subregional stratified analysis revealed notable geographic heterogeneity in the associations between social vulnerability and long COVID incidence. For example, disability prevalence consistently showed positive associations in the Midwest, South, and Southwest, whereas the protective effect of vaccination coverage was largely confined to the Midwest and South. Minoritised population share exhibited opposite directions of association across regions positive in the Midwest and Southwest but negative in the South and West, highlighting region-specific social and structural contexts. Limited English proficiency became a significant predictor of higher incidence only after the Omicron period in the Midwest and Southwest. These findings underscore that the social determinants of long COVID were not uniform across the United States but are shaped by localized socioeconomic, cultural, and healthcare factors, emphasizing the need for regionally tailored public health strategies.

Given the significant spatiotemporal disparities in long COVID incidence, policy responses should promote equitable diagnosis and care. Targeted healthcare resource allocation with risk stratification is essential, with high-incidence areas requiring specialized long COVID clinics, rehabilitation centers, and increased funding for chronic disease prevention and control, while low-incidence areas should be assessed for under-reporting, under-diagnosis, clinician competency, and population awareness gaps.^37^ Given that many high-risk regions are in underserved Midwestern areas, expanding telemedicine services and deploying mobile health units can improve accessibility to specialized care for acute COVID-19, long COVID, and existing comorbidities.^38^ Additionally, in areas with limited healthcare facilities and low population engagement with the healthcare system for long COVID diagnosis and care, outreach programs and social support initiatives should be implemented to improve early detection and intervention and reduce the entrenched individual-level and social determinants of health inequities. Related policies, such as the disability measure,^39^ can also be adjusted to reflect geographic and demographic disparities, ensuring adequate support for long COVID patients facing economic and healthcare barriers.^37^ Addressing these disparities through policy-driven health interventions, including healthcare accessibility improvements and targeted public health interventions implementation will help mitigate the short-term and long-term impact of long COVID, ultimately leading to better health outcomes for vulnerable populations, reduced associated health costs for the governments, and improved health equity.

This study has several limitations. First, due to constraints in the N3C EHR database, approximately 35% of long COVID records lacked corresponding COVID-19 EHR records, likely because many patients experienced mild symptoms and did not seek healthcare, leading to a dataset biased toward individuals with more severe acute infections.^40^ Second, consistent with data from most surveillance systems, the long COVID incidence estimated from N3C sentinel surveillance system might not represent the true burden of the disease due to under-ascertainment bias from symptomatic cases who do not seek health care due to healthcare access (physical and/or economic) constraints and poor awareness of long COVID symptoms. Third, sampling bias may have been introduced due to the limited number of sentinel sites included in the database, and another important limitation is the uneven distribution of these sites across U.S. regions, which may have led to some areas being oversampled while others were under-sampled. This imbalance could introduce regional biases in the data, potentially affecting observed incidence rates and risk factor associations, thereby reducing the generalizability of the findings to the broader U.S. population, particularly in regions with fewer or no sentinel sites where healthcare access and demographic characteristics may differ significantly. Fourth, our method for estimating incidence risks involved excluding patients who had not received a long COVID diagnosis within 180 days after their COVID-19 infection, which may have led to measurement bias resulting from an underestimation of the actual disease burden. Moreover, it is also possible that the differences in the data collection time period for the N3C EHR and SVI databases have contributed to measurement bias. Fifth, due to uneven data collection in the N3C EHR system, regions with well-resourced healthcare facilities may report higher long COVID diagnoses due to improved detection from robust screening and diagnostic practices, while under-resourced areas may underreport cases, potentially distorting geographic patterns. Additionally, changes in diagnostic awareness and testing practices for long COVID over time may have influenced observed temporal patterns of long COVID incidence. Early in the pandemic, limited clinical recognition and inconsistent diagnostic coding could have led to under-detection, while increased awareness and the establishment of long COVID clinics in later periods may have improved case ascertainment. Furthermore, the definition of long COVID itself evolved over time, as diagnostic criteria and clinical understanding became more standardized, which may also have affected case identification and comparability across time periods. These temporal shifts in testing and diagnostic behavior were not explicitly captured in this analysis.^41^ Sixth, given the sparse spatial distribution of reported long COVID cases across the U.S., we conducted analyses at the county level rather than the ZIP code level to ensure robust and stable incidence estimates, and excluded counties with fewer than 20 COVID-19 records. However, this aggregation may have reduced spatial precision and masked within-county variability, potentially affecting the detection of small-scale spatial correlation. Finally, since long COVID is an extremely heterogeneous condition encompassing a wide range of symptoms,^42^ our study did not differentiate cases by specific symptom profiles, which may obscure important subgroup trends. Future research should explore symptom-specific patterns and pre-existing medical conditions to enhance the understanding of long COVID incidence and its spatiotemporal patterns and wider social determinants of health for improved design and delivery of tailored and targeted health and social interventions at both the population and healthcare system levels.

## Contributors

Z. Chen and B. Li had full access to all the data in the study and took responsibility for the integrity of the data and the accuracy of the data analysis.

*Concept and design:* Z. Chen, B. Li, Y. Chen, J. Liu, F. Luo, X. Chen, Y. Shen.

*Acquisition, analysis, or interpretation of data:* Z. Chen, B. Li, Y. Chen, J. Liu, F. Luo.

*Drafting of the manuscript:* Z. Chen, B. Li, Y. Chen, J. Liu, F. Luo.

*Critical revision of the manuscript for important intellectual content:* Z. Chen, B. Li, Y. Chen, J. Liu, F. Luo, K. Ogunyemi, Y. Ge, Y. Ke, Y. Yang, X. Chen, Y. Shen.

*Statistical analysis:* Z. Chen, B. Li, Y. Chen.

*Obtained funding:* Y. Shen.

*Administrative, technical, or material support:* X. Chen, Y. Shen.

*Supervision:* X. Chen, Y. Shen.

## Data sharing statement

All data used in this study is available through the N3C Enclave to approved users. See https://covid.cd2h.org/for-researchers for instructions on how to access the data. The analytic code is publicly available on GitHub (https://github.com/Zhetao-Chen/Long-Covid).

## Missing maps disclaimer

In the related maps, counties indicated in white represent areas excluded from the analysis based on the predefined analytic criteria described in Section 2 due to insufficient data availability.

## Editor note

The Lancet Group takes a neutral position with respect to territorial claims in published maps and institutional affiliations.

## Declaration of interests

All authors report no potential conflicts of interest.

## Appendix A Supplementary data

Supplementary Material

Collaborating Group
