# Supplementary material for: Mapping spatial and social inequities of long COVID across the United States: a retrospective cohort study
Source: Lancet Reg Health Am. 2026 Feb 13;56:101401. doi: 10.1016/j.lana.2026.101401 (PMC12925177; doi:10.1016/j.lana.2026.101401)
Supplement: Supplementary Material [file mmc1.docx]

**Supplementary Material:** Mapping spatial and social inequities of long COVID across the United States: a retrospective cohort study

Zhetao Chen^1†^, Bingnan Li^2†^, Yewen Chen^1†^, Jialing Liu^1^, Fangzhi Luo^1^, Kehinde Olawale Ogunyemi^1^, Yang Ge^1^, Yuan Ke^2^, Yang Yang^2^, Xianyan Chen^1*^, Ye Shen^1*^, on behalf of N3C consortium

**Affiliations:**

^1^Epidemiology & Biostatistics, College of Public Health, The University of Georgia, Athens, GA, USA

^2^Department of Statistics, Franklin College of Arts and Science, The University of Georgia, Athens, GA, USA

† The first three authors contribute equally to this article

* **Correspondence**:

X. Chen: [xychen@uga.edu](mailto:xychen@uga.edu)

Y. Shen: yeshen@uga.edu

# S1 EHR of long COVID and data processing

Given the inherent characteristics of electronic health record (EHR) data, which primarily capture individuals with more severe symptoms who seek in-person medical care, our long COVID cohort is likely biased toward patients experiencing more severe or persistent symptoms. Notably, approximately 40% of identified long COVID cases lack corresponding acute COVID-19 records in the EHR, likely due to patients experiencing mild or asymptomatic acute infections and recovering at home without medical intervention, resulting in an absence of documented acute disease. As our objective is to estimate and compare the incidence risk of long COVID across regions, we define the at-risk population as individuals with a confirmed acute COVID-19 diagnosis, allowing for standardized denominator calculations. long COVID cases without a documented acute infection are therefore excluded to ensure the validity of risk estimation. This exclusion is essential for minimizing misclassification bias, as the absence of an acute COVID-19 record precludes accurate determination of symptom onset relative to infection, leading to potential inflation or deflation of risk estimates. Additionally, these cases may represent individuals who sought care only for persistent symptoms rather than those with a well-defined transition from acute infection to long COVID, introducing heterogeneity that could bias comparisons across regions. Furthermore, the completeness of acute COVID-19 documentation varies across healthcare systems, with differences in testing accessibility and healthcare-seeking behaviors influencing EHR records. By restricting the cohort to individuals with documented acute infections, we enhance the comparability of risk estimates across populations and mitigate potential confounding arising from regional variations in EHR capture. This approach ensures a robust and interpretable assessment of long COVID incidence following acute infection.

For identifying long COVID cases, we utilized ICD-10 codes B94.8 and U09.9 as diagnostic indicators. Since U09.9 was officially introduced as the long COVID diagnosis code on October 1, 2021, diagnoses related to long COVID prior to this date predominantly used the B94.8 code. Therefore, we applied a temporal cutoff on October 1, 2021, selecting EHR records with B94.8 diagnoses before this date and U09.9 diagnoses thereafter. This approach ensures comprehensive coverage of long COVID cases across different time periods, accounting for the evolution of diagnostic practices.

We initiated data cleaning using the original N3C dataset. Overall cleaning process was summarized in **Figure S1**. On one hand, COVID-19 patient records were cleaned, and on the other, long COVID patient records were similarly processed. Only records corresponding to a patient’s first COVID-19 infection or first long COVID infection were retained. In the process of filtering N3C EHR data, we also applied site-level selection criteria. During data cleaning, we observed that certain sites had ceased their collaboration with N3C by the time long COVID cases began to emerge. Including EHR data from these sites could potentially bias our analysis by underestimating the incidence of long COVID, thereby affecting subsequent data interpretation. To ensure data quality and consistency, we excluded sites with a long COVID incidence rate below 0.1%, as we deemed these sites invalid for analysis due to insufficient case reporting or incomplete data coverage. This site-level filtering helps improve the robustness of our incidence estimates and ensures more reliable regional comparisons.


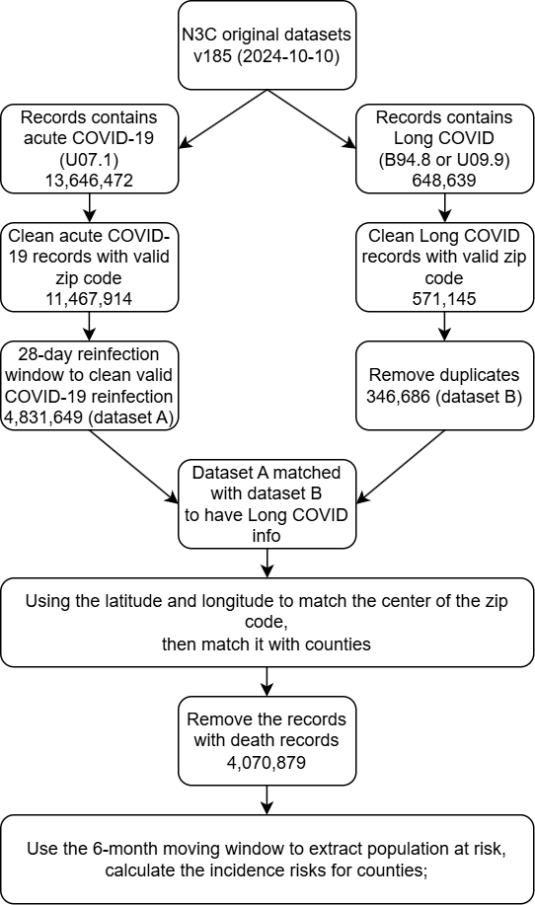


**Figure S1**: Diagram of data filtering and cleaning.

# S2 Dynamic incidence risk and definition of long COVID incidence

It is important to emphasize that all long COVID incidence calculations in this study are based exclusively on individuals who were previously diagnosed with acute COVID-19. That is, the at-risk population for all incidence metrics consistently includes only those with documented acute COVID-19. For the dynamic incidence risk algorithm shown in **Figure S2**, the criteria for defining the dynamic population at risk are as follows:

1. **Earliest inclusion date for the population at risk:** We determined the start of the population at risk as 180 days prior to the earliest date of the target period. For example, for long COVID cases identified between January 1, 2021, and March 31, 2021, the corresponding population at risk consists of COVID-19 patients recorded in the EHR starting from July 1, 2020.
2. **Latest inclusion date for the population at risk:** Following CDC guidelines, we adopted a one-month timeframe as the allowable window between the COVID-19 diagnosis and the long COVID diagnosis. The study [Crowd-sourced machine learning prediction of long COVID using data from the National COVID Cohort Collaborative] suggests that even when using B94.8 and U09.9 codes, a more conservative approach can be applied by using a 28-day threshold as a filter to refine long COVID data.


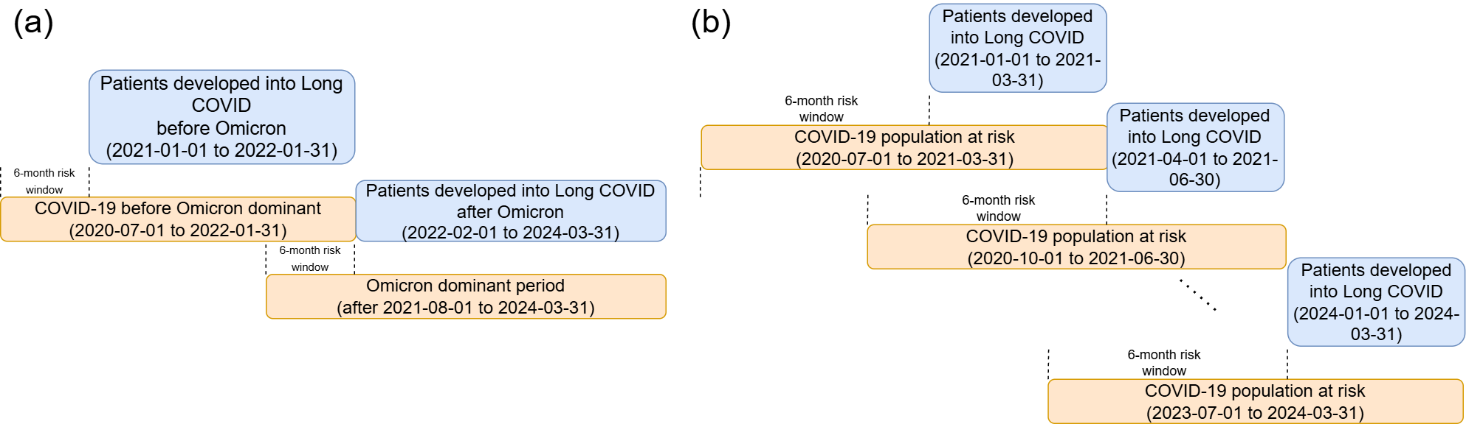


**Figure S2**: Dynamic incidence risk calculation. (a) The incidence of long COVID before and after the emergence of the Omicron variant in January 2022; and (b) The quarterly incidence of long COVID between 2021 and 2024.

In calculating these outcomes, we set a threshold of 180 days as a risk window to calculate dynamic incidence risk. This choice was made based on our observations from the N3C long COVID EHR and previous findings^1^, where most long COVID cases (over 77%) following an acute COVID-19 infection were diagnosed within 180 days of post-infection (**Figure S3** of the Supplementary Material). If a patient was not diagnosed with long COVID within this threshold, we considered them no longer at risk of developing long COVID. Based on the guidance on the International Classification of Diseases-10th Revision-Clinical Modification (ICD-10-CM), we identified acute COVID-19 using the U07.1 code to match our long-COVID definition, which relies on the B94.8 or U09.9 codes because long COVID has no corresponding biomarker tests^2^. To ensure a robust estimation of long COVID incidence during the dynamic incidence risk calculation, each time interval of interest was required to include at least 20 COVID-19 cases^3^.


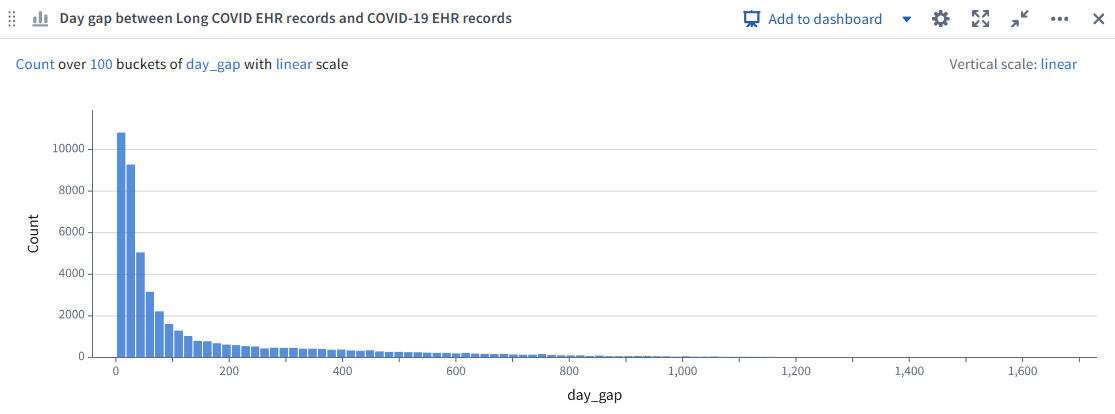
**Figure S3**: Summary histogram of gap days between acute COVID-19 diagnosis and long COVID diagnosis

We plot the distribution of long COVID cases (**Figure S4**), which is highly uneven across counties, with large portions of the country reporting only a small number of cases. Even at this geographic scale, reporting remains limited: only 1,063 out of 3,144 counties had at least one documented long COVID case and were therefore included in the incidence analysis. Conducting analyses at a finer spatial resolution (e.g., ZIP code) would further exacerbate data sparsity, resulting in unstable estimates driven by small population sizes and inconsistent reporting coverage.

**Figure S4**: The Distribution of Long COVID Cases in U.S. Counties

# S3 Data exploration analysis

For each county $i$, the local Moran’s I was computed as:

$I_{i}=\frac{\left( x_{i}-\overline{x} \right)}{S^{2}}\sum_{j=1}^{N} w_{ij}(x_{j}-\overline{x})$,

where $N=673$ is the total number of U.S. counties, $x_{i}$ is the incidence in county $i$, $\overline{x}$ is the average incidence across counties, $S^{2}$ is the variance of incidences, and $w_{ij}$ is the spatial weight between counties $i$ and $j$. The weights were defined based on semivariogram analysis results using an exponential function (**Figure** **S5**). Statistical significance for both methods was assessed using a Monte Carlo simulation by generating a pseudo-random distribution to determine whether the observed Moran’s I and Getis is significantly different from randomness^4^.

**Figure S5:** Semi-variogram analysis of county mean incidence before and after Omicron


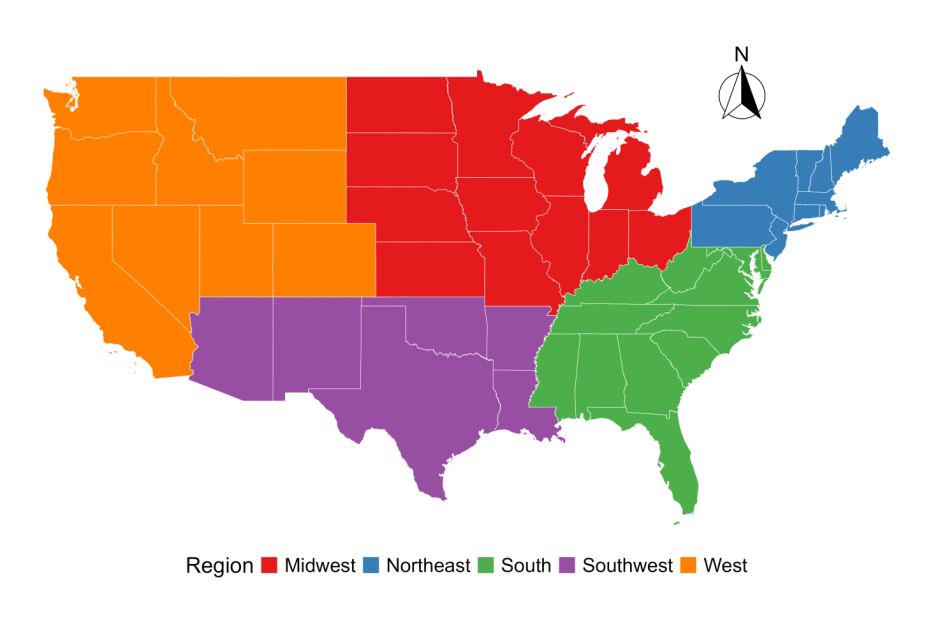


**Figure S6**: Five-subregion division of the United States

# S4 Long COVID incidence patterns incidence before and after Omicron dominance across different subregions of the United States

**Table S1**. Definitions of candidate covariates included in long COVID incidence modeling.

| **No.** | **Full Name** | **Definition** | **Source** |
| --- | --- | --- | --- |
| 1 | Vaccination^1,2^ | First-dose COVID-19 vaccination rate | CDC |
| 2 | RUCC^1,2^ | Rural-Urban Continuum Codes | USDA |
| 3 | POV150^2^ | Percentage of persons below 150% poverty estimate | SVI |
| 4 | MOBILE^1^ | Percentage of mobile homes estimate | SVI |
| 5 | UNINSUR | Percentage uninsured in the total civilian noninstitutionalized population estimate | SVI |
| 6 | NOVEH^1^ | Percentage of households with no vehicle available estimate | SVI |
| 7 | CROWD^1^ | Percentage of occupied housing units with more people than rooms estimate | SVI |
| 8 | UNEMP | Unemployment Rate estimate | SVI |
| 9 | DISABL^1,2^ | Percentage of civilian noninstitutionalized population with a disability estimate | SVI |
| 10 | SNGPNT | Percentage of single-parent households with children under 18 estimates | SVI |
| 11 | MUNIT^2^ | Percentage of housing in structures with 10 or more units estimate | SVI |
| 12 | GROUPQ^1,2^ | Percentage of persons in group quarters estimate | SVI |
| 13 | HBURD^1^ | Percentage of housing cost-burdened occupied housing units with annual income less than $75,000 (30%+ of income spent on housing costs) estimate | SVI |
| 14 | NOHSDP^1,2^ | Percentage of persons with no high school diploma (age 25+) estimate | SVI |
| 15 | AGE65^2^ | Percentage of persons aged 65 and older estimate | SVI |
| 16 | AGE17 | Percentage of persons aged 17 and younger estimate | SVI |
| 17 | LIMENG^2^ | Percentage of persons (age 5+) who speak English "less than well" estimate | SVI |
| 18 | MINRTY^1,2,3^ | Percentage minority | SVI |

^1^Selected in the pre-Omicron stepwise regression model;

^2^Selected in the post-Omicron stepwise regression model;

^3^Minority population includes (based on the U.S. Census Bureau’s American Community Survey): Hispanic or Latino (of any race); Black and African American (Not Hispanic or Latino); American Indian and Alaska Native (Not Hispanic or Latino); Asian (Not Hispanic or Latino); Native Hawaiian and Other Pacific Islander (Not Hispanic or Latino); Two or More Races (Not Hispanic or Latino); and Other Races (Not Hispanic or Latino).

**Table S2.** Changes in the number of counties with long COVID incidence before and after Omicron dominance across different subregions of the United States (n = 673 counties).

| Region | The number of counties | | | |
| --- | --- | --- | --- | --- |
|  | Decreased | Increased | No Change | Increased Rate^a^ (%) |
| Northeast | 15 | 45 | 0 | 75.00 |
| West | 27 | 63 | 0 | 70.00 |
| Midwest | 72 | 165 | 0 | 69.62 |
| Southwest | 11 | 23 | 0 | 67.65 |
| South | 83 | 168 | 1 | 66.67 |
| Total | 208 | 464 | 1 | 68.95 |

^a^Increased Rate = Increased$\times$100 / (Increased + Decreased + No Change).

**Table S3**: Average of long COVID incidence before and after Omicron across different subregions of the United States.

| Region | Average incidence across counties | |
| --- | --- | --- |
|  | Before (%) | After (%) |
| Northeast | 1.66 | 2.45 |
| West | 2.42 | 2.86 |
| Midwest | 2.23 | 2.79 |
| Southwest | 1.85 | 1.78 |
| South | 2.21 | 2.53 |

**Figure S7**: The map for the difference in average incidences between before and after the dominance of Omicron (i.e., January 2022).

**Table S4.** Regional comparison of significant local Moran’s I before and after Omicron (n = 673 counties).

| Region | The number (rate^a^) of counties with positive correlation | |  | The number (rate) of counties with negative correlation | |
| --- | --- | --- | --- | --- | --- |
|  | Before | After |  | Before | After |
| South | 130 (51.6%) | 128 (50.8%) |  | 37 (14.7%) | 36 (14.3%) |
| Northeast | 26 (43.3%) | 13 (21.7%) |  | 5 (8.3%) | 3 (5.0%) |
| Midwest | 54 (22.8%) | 55 (23.2%) |  | 17 (7.2%) | 30 (12.7%) |
| West | 10 (11.1%) | 29 (32.2%) |  | 10 (11.1%) | 18 (20.0%) |
| Southwest | 0 (0.0%) | 13 (38.2%) |  | 4 (11.8%) | 3 (8.8%) |
| Total | 220 (32.7%) | 238 (35.4%) |  | 73 (10.8%) | 90 (13.4%) |

^a^ Rate = (Number of significant counties) $\times$ 100 / (Total number of counties in the region).

**Figure S8**: Spatial correlation of long COVID incidence across U.S. counties assessed using local Moran’s I: (a) before and (b) after Omicron dominance.

# S5 Stepwise Regression

We first adopt stepwise regression model to identify a subset of variables that best explain the variation in our study outcome, i.e., incidence of long COVID. To stable variance of incidence, we employed a logarithmic transformation for incidence (**Figure S9**). To account for potential heterogeneity across different pandemic phases, we performed the stepwise variable selection based on the Akaike Information Criterion (AIC) separately for the pre-Omicron and post-Omicron periods. This approach allowed us to identify distinct sets of variables that were most predictive within each time frame. The results from stepwise regression were present to the following **Tables S5-S6**. The selected set did not concentrate redundant, highly correlated variables (**Figure S10**). Aside from a small number of expected pairs — pre-Omicron: Urban–Rural Status (urban) with Disabled Population, and Housing Cost Burden with Minority Population; post-Omicron: Below 150% Poverty Level with No High School Diploma, Minority Population with Limited English Proficiency, and Disabled Population with Below 150% Poverty Level — pairwise correlations among retained predictors were generally modest. This indicates that multicollinearity was largely addressed by the selection, and any residual collinearity is further mitigated by our Bayesian hierarchical specification (with weakly informative priors and spatial random effects), which stabilizes estimation and inference. According to the variables selected in each period, we subsequently fitted two separate regression models. For clarity, variables that were consistently selected in both periods are presented together (**Figure 3**), while those uniquely identified in either the pre- or post-Omicron analyses are displayed separately (**Figure S12**). Furthermore, we compared the above results with an additional analysis using the full set of 18 covariables without any variable selection (**Figure S13**). Reassuringly, the direction of most associations remained consistent with the stepwise-selected models, indicating that the key inferences were not driven by variable selection artifacts. However, several notable differences emerged. The associations for minority population share and housing cost burden were no longer statistically significant in the pre-Omicron period, and the proportion of group-quarters residents was no longer significant in either period. In contrast, crowded housing, which was excluded by stepwise selection, became a significant negative predictor of long COVID incidence in the post-Omicron period (–0.051, –0.101 to –0.002).

**Figure S9**: Distribution of county-level long COVID incidence before (a) and after (b) Omicron dominance, and the corresponding log-transformed counterparts, (c) and (d).

**Table S5.** Stepwise regression results for long COVID incidence before Omicron dominance.

| Estimate  (95% CI)  p-value | Model 1 | Model 2 | Model 3 | Model 4 | Model 5 |
| --- | --- | --- | --- | --- | --- |
| Disabled Population^a^ | 0.046  (0.035, 0.058)  <.001* | 0.037  (0.024, 0.049)  <.001* | 0.036  (0.023, 0.049)  <.001* | 0.047  (0.031, 0.062)  <.001* | 0.045  (0.026, 0.064)  <.001* |
| Minority Population^b^ | -0.006  (-0.009, -0.004)  <.001* | -0.008  (-0.011, -0.005)  <.001* | -0.007  (-0.010, -0.004)  <.001* | -0.004  (-0.008, -0.001)  0.024* | -0.005  (-0.009, -0.001)  0.008* |
| Urban-Rural Status (Urban) | —— | -0.222  (-0.331, -0.114)  <.001* | -0.201  (-0.311, -0.091)  <.001* | -0.203  (-0.315, -0.091)  <.001* | -0.193  (-0.305, -0.080)  <.001* |
| Housing Cost Burden | —— | 0.017  (0.005, 0.028)  0.004* | 0.024  (0.012, 0.036)  <.001* | 0.020  (0.008, 0.032)  0.001* | 0.020  (0.008, 0.033)  0.002* |
| No Vehicle Available | —— | —— | -0.017  (-0.028, -0.005)  0.005* | -0.015  (-0.027, -0.003)  0.013* | -0.013  (-0.025, -0.001)  0.029* |
| First Vaccination Rate | —— | —— | -0.004  (-0.008, -0.001)  0.016* | -0.005  (-0.009, -0.001)  0.007* | -0.006  (-0.009, -0.002)  0.004* |
| No High School Diploma | —— | —— | —— | -0.018  (-0.032, -0.003)  0.015* | -0.029  (-0.046, -0.011)  0.001* |
| Group Quarters | —— | —— | —— | 0.015  (0.001, 0.030)  0.036* | 0.015  (0.000, 0.030)  0.044* |
| Crowded Housing | —— | —— | —— | —— | 0.033  (-0.008, 0.075)  0.117 |
| Mobile Homes | —— | —— | —— | —— | 0.006  (-0.002, 0.014)  0.122 |

^a^ Disability refers to the proportion of the civilian noninstitutionalized population with any disability; ^b^ The minority status refers to the percentage of the population that is not non-Hispanic White.

**Table S6.** Stepwise regression results for long COVID incidence after Omicron dominance.

| OR (95%CI) p-value | Model 1 | Model 2 | Model 3 | Model 4 | Model 5 |
| --- | --- | --- | --- | --- | --- |
| Minority Population | -0.012 (-0.015, -0.010) <.001* | -0.010 (-0.012, -0.007) <.001* | -0.009 (-0.012, -0.006) <.001* | -0.009 (-0.013, -0.006) <.001* | -0.010 (-0.013, -0.006) <.001* |
| Below 150% Poverty Level | 0.015 (0.009, 0.021) <.001* | 0.018 (0.010, 0.027) <.001* | 0.012 (0.002, 0.023) 0.020* | 0.013 (0.002, 0.023) 0.019* | 0.014 (0.003, 0.025) 0.011* |
| Urban-Rural Status (Urban) | —— | -0.160 (-0.264, -0.056) 0.003* | -0.187 (-0.293, -0.080) <.001* | -0.173 (-0.280, -0.067) 0.001* | -0.141 (-0.249, -0.033) 0.010* |
| No High School Diploma | —— | -0.019 (-0.032, -0.006) 0.003* | -0.023 (-0.036, -0.010) <.001* | -0.039 (-0.055, -0.023) <.001* | -0.047 (-0.064, -0.030) <.001* |
| Population Age 65+ | —— | —— | -0.017 (-0.030, -0.005) 0.008* | -0.017 (-0.029, -0.004) 0.011* | -0.019 (-0.032, -0.006) 0.005* |
| Disabled Population^b^ | —— | —— | 0.021 (0.003, 0.040) 0.023* | 0.031 (0.012, 0.051) 0.002* | 0.031 (0.011, 0.050) 0.002* |
| Limited English | —— | —— | —— | 0.039 (0.011, 0.068) 0.007* | 0.056 (0.026, 0.087) <.001* |
| First Vaccination Rate | —— | —— | —— | -0.005 (-0.009, -0.001) 0.010* | -0.004 (-0.008, 0.000) 0.052 |
| Group Quarters | —— | —— | —— | —— | 0.016 (0.002, 0.030) 0.025* |
| Multi-Unit Housing | —— | —— | —— | —— | -0.012 (-0.021, -0.003) 0.009* |

**Figure S10**: Correlation matrices of social determinants before (left) and after (right) the Omicron period. **^1^**Selected in the pre-Omicron stepwise regression model; **^2^**Selected in the post-Omicron stepwise regression model.

# S6 Spatial random effect models

**Figure S11**: Comparison of residual distributions before and after incorporating spatial random effects in models fitted before and after Omicron dominance: (a) No spatial effect – before Omicron; (b) Spatial effect – before Omicron; (c) No spatial effect – after Omicron; (d) Spatial effect – after Omicron. The “Correlation” value reported in each panel denotes the Pearson correlation between the fitted values from the model and the observed outcome.

**Figure S12**: Spatial random effect models yielded posterior estimates (95% credible intervals (CIs)) of variables uniquely selected before (a) and after (b) Omicron dominance.

**Figure S13**: Posterior Estimates (95% credible intervals (CIs)) of All Social Determinants from Spatial Random Effects Models Before and After Omicron Dominance

**Figure S14**: Posterior Estimates (95% credible intervals (CIs)) from Spatial Random Effects Models Using the Overall Social Vulnerability Score Before and After Omicron Dominance

**Figure S15**: Regional Posterior Effect Estimates (95% credible intervals (CIs)) of Social Vulnerability Determinants on Long COVID from Bayesian Spatial Random Effects Models Before and After Omicron Dominance

**Figure S16**: Regional Posterior Estimates (95% Credible Intervals) from Spatial Random Effects Models Using the Overall Social Vulnerability Score Before and After Omicron Dominance

**Table S7.** Regional differences in long COVID incidence by social vulnerability factors thresholds

| Variable | Region | Average incidence across counties (%) | | | |
| --- | --- | --- | --- | --- | --- |
|  |  | Level ≤ Threshold | Level > Threshold | Difference | p value |
| Disability Status Group (threshold: 10%) | Midwest | 0.87 | 1.62 | 0.75 | <0.0001 |
|  | Northeast | 0.75 | 1.45 | 0.7 | <0.0001 |
|  | South | 0.61 | 1.46 | 0.85 | <0.0001 |
|  | Southwest | 1.33 | 1.13 | -0.2 | 0.0687 |
|  | West | 1.32 | 1.99 | 0.67 | 0.0007 |
| First Vaccine Rate (threshold: 60%) | Midwest | 1.85 | 1.27 | -0.58 | <0.0001 |
|  | Northeast | 1.38 | 1.33 | -0.05 | 0.0254 |
|  | South | 1.75 | 1.2 | -0.55 | <0.0001 |
|  | Southwest | 1.26 | 0.99 | -0.27 | 0.0994 |
|  | West | 2.99 | 1.58 | -1.41 | <0.0001 |
| Group Quarters Population (threshold: 3%) | Midwest | 1.48 | 1.71 | 0.23 | 0.0166 |
|  | Northeast | 1.27 | 1.41 | 0.14 | 0.0299 |
|  | South | 1.34 | 1.58 | 0.24 | <0.0001 |
|  | Southwest | 1.11 | 1.25 | 0.14 | 0.1059 |
|  | West | 1.89 | 1.82 | -0.07 | 0.9177 |
| Minority Population (threshold: 20%) | Midwest | 1.61 | 1.37 | -0.24 | 0.0006 |
|  | Northeast | 1.6 | 0.91 | -0.69 | <0.0001 |
|  | South | 1.64 | 1.21 | -0.43 | <0.0001 |
|  | Southwest | 1.28 | 1.13 | -0.15 | 0.1022 |
|  | West | 2.22 | 1.59 | -0.63 | <0.0001 |
| Population Below 150% Poverty  (threshold: 12%) | Midwest | 0.98 | 1.61 | 0.63 | <0.0001 |
|  | Northeast | 0.89 | 1.43 | 0.54 | 0.0003 |
|  | South | 0.7 | 1.46 | 0.76 | <0.0001 |
|  | Southwest | 1.69 | 1.13 | -0.56 | 0.0219 |
|  | West | 1.59 | 1.9 | 0.31 | 0.1979 |
| Rural-Urban Category (Urban: Rural) | Midwest | 1.47 | 1.73 | 0.26 | <0.0001 |
|  | Northeast | 1.2 | 1.68 | 0.48 | <0.0001 |
|  | South | 1.17 | 1.98 | 0.81 | <0.0001 |
|  | Southwest | 1.03 | 1.65 | 0.62 | <0.0001 |
|  | West | 1.6 | 2.76 | 1.16 | <0.0001 |

**Reference**

1. Bowe B, Xie Y, Al-Aly Z. Postacute sequelae of COVID-19 at 2 years. *Nature medicine.* 2023;29(9):2347-2357.

2. ICD10data.com. 2024 ICD-10-CM diagnosis code U09. 2024; <https://www.icd10data.com/ICD10CM/Codes/U00-U85/U00-U49/U09-/U09.9>. Accessed January 1, 2025.

3. Parker J, Talih M, Malec DJ, et al. National Center for Health Statistics data presentation standards for proportions. 2017.

4. Anselin L. Local indicators of spatial association—LISA. *Geographical analysis.* 1995;27(2):93-115.
