# Supplementary material for: Mapping spatial and social inequities of long COVID across the United States: a retrospective cohort study
Source: Lancet Reg Health Am. 2026 Feb 13;56:101401. doi: 10.1016/j.lana.2026.101401 (PMC12925177; doi:10.1016/j.lana.2026.101401)
Supplement: Collaborating Group [file mmc2.docx]

| **Full Name** | **Institutional Affiliation** |
| --- | --- |
| Adam B. Wilcox | National COVID Cohort Collaborative (N3C) |
| Adam M. Lee | National COVID Cohort Collaborative (N3C) |
| Alexis Graves | National COVID Cohort Collaborative (N3C) |
| Alfred (Jerrod) Anzalone | National COVID Cohort Collaborative (N3C) |
| Amin Manna | National COVID Cohort Collaborative (N3C) |
| Amit Saha | National COVID Cohort Collaborative (N3C) |
| Amy Olex | National COVID Cohort Collaborative (N3C) |
| Andrea Zhouss | National COVID Cohort Collaborative (N3C) |
| Andrew E. Williams | National COVID Cohort Collaborative (N3C) |
| Andrew M. Southerland | National COVID Cohort Collaborative (N3C) |
| Andrew T. Girvin | National COVID Cohort Collaborative (N3C) |
| Anita Walden | National COVID Cohort Collaborative (N3C) |
| Anjali Sharathkumar | National COVID Cohort Collaborative (N3C) |
| Benjamin Amor | National COVID Cohort Collaborative (N3C) |
| Benjamin Bates | National COVID Cohort Collaborative (N3C) |
| Brian Hendricks | National COVID Cohort Collaborative (N3C) |
| Brijesh Patel | National COVID Cohort Collaborative (N3C) |
| G. Caleb Alexander | National COVID Cohort Collaborative (N3C) |
| Carolyn T. Bramante | National COVID Cohort Collaborative (N3C) |
| Cavin Ward-Caviness | National COVID Cohort Collaborative (N3C) |
| Charisse Madlock-Brown | National COVID Cohort Collaborative (N3C) |
| Christine Suver | National COVID Cohort Collaborative (N3C) |
| Christopher G. Chute | National COVID Cohort Collaborative (N3C) |
| Christopher Dillon | National COVID Cohort Collaborative (N3C) |
| Chunlei Wu | National COVID Cohort Collaborative (N3C) |
| Clare Schmitt | National COVID Cohort Collaborative (N3C) |
| Cliff Takemoto | National COVID Cohort Collaborative (N3C) |
| Dan Housman | National COVID Cohort Collaborative (N3C) |
| Davera Gabriel | National COVID Cohort Collaborative (N3C) |
| David A. Eichmann | National COVID Cohort Collaborative (N3C) |
| Diego Mazzotti | National COVID Cohort Collaborative (N3C) |
| Donald E. Brown | National COVID Cohort Collaborative (N3C) |
| Eilis Boudreau | National COVID Cohort Collaborative (N3C) |
| Elaine L. Hill | National COVID Cohort Collaborative (N3C) |
| Emily Carlson Marti | National COVID Cohort Collaborative (N3C) |
| Emily R. Pfaff | National COVID Cohort Collaborative (N3C) |
| Evan French | National COVID Cohort Collaborative (N3C) |
| Farrukh M Koraishy | National COVID Cohort Collaborative (N3C) |
| Federico Mariona | National COVID Cohort Collaborative (N3C) |
| Fred Prior | National COVID Cohort Collaborative (N3C) |
| George Sokos | National COVID Cohort Collaborative (N3C) |
| Greg Martin | National COVID Cohort Collaborative (N3C) |
| Harold P. Lehmann | National COVID Cohort Collaborative (N3C) |
| Heidi Spratt | National COVID Cohort Collaborative (N3C) |
| Hemalkumar B. Mehta | National COVID Cohort Collaborative (N3C) |
| J.W. Awori Hayanga | National COVID Cohort Collaborative (N3C) |
| Jami Pincavitch | National COVID Cohort Collaborative (N3C) |
| Jaylyn Clark | National COVID Cohort Collaborative (N3C) |
| Jeremy Richard Harper | National COVID Cohort Collaborative (N3C) |
| Jessica Yasmine Islam | National COVID Cohort Collaborative (N3C) |
| Jin Ge | National COVID Cohort Collaborative (N3C) |
| Joel Gagnier | National COVID Cohort Collaborative (N3C) |
| Johanna J. Loomba | National COVID Cohort Collaborative (N3C) |
| John B. Buse | National COVID Cohort Collaborative (N3C) |
| Jomol Mathew | National COVID Cohort Collaborative (N3C) |
| Joni L. Rutter | National COVID Cohort Collaborative (N3C) |
| Julie A. McMurry | National COVID Cohort Collaborative (N3C) |
| Justin Guinney | National COVID Cohort Collaborative (N3C) |
| Justin Starren | National COVID Cohort Collaborative (N3C) |
| Karen Crowley | National COVID Cohort Collaborative (N3C) |
| Katie Rebecca Bradwell | National COVID Cohort Collaborative (N3C) |
| Kellie M. Walters | National COVID Cohort Collaborative (N3C) |
| Ken Wilkins | National COVID Cohort Collaborative (N3C) |
| Kenneth R. Gersing | National COVID Cohort Collaborative (N3C) |
| Kenrick Cato | National COVID Cohort Collaborative (N3C) |
| Kimberly Murray | National COVID Cohort Collaborative (N3C) |
| Kristin Kostka | National COVID Cohort Collaborative (N3C) |
| Lavance Northington | National COVID Cohort Collaborative (N3C) |
| Lee Pyles | National COVID Cohort Collaborative (N3C) |
| Lesley Cottrell | National COVID Cohort Collaborative (N3C) |
| Lili M. Portilla | National COVID Cohort Collaborative (N3C) |
| Mariam Deacy | National COVID Cohort Collaborative (N3C) |
| Mark M. Bissell | National COVID Cohort Collaborative (N3C) |
| Marshall Clark | National COVID Cohort Collaborative (N3C) |
| Mary Emmett | National COVID Cohort Collaborative (N3C) |
| Matvey B. Palchuk | National COVID Cohort Collaborative (N3C) |
| Melissa A. Haendel | National COVID Cohort Collaborative (N3C) |
| Meredith Adams | National COVID Cohort Collaborative (N3C) |
| Meredith Temple-O’Connor | National COVID Cohort Collaborative (N3C) |
| Michael G. Kurilla | National COVID Cohort Collaborative (N3C) |
| Michele Morris | National COVID Cohort Collaborative (N3C) |
| Nasia Safdar | National COVID Cohort Collaborative (N3C) |
| Nicole Garbarini | National COVID Cohort Collaborative (N3C) |
| Noha Sharafeldin | National COVID Cohort Collaborative (N3C) |
| Ofer Sadan | National COVID Cohort Collaborative (N3C) |
| Patricia A. Francis | National COVID Cohort Collaborative (N3C) |
| Penny Wung Burgoon | National COVID Cohort Collaborative (N3C) |
| Philip R.O. Payne | National COVID Cohort Collaborative (N3C) |
| Randeep Jawa | National COVID Cohort Collaborative (N3C) |
| Rebecca Erwin-Cohen | National COVID Cohort Collaborative (N3C) |
| Rena C. Patel | National COVID Cohort Collaborative (N3C) |
| Richard A. Moffitt | National COVID Cohort Collaborative (N3C) |
| Richard L. Zhu | National COVID Cohort Collaborative (N3C) |
| Rishikesan Kamaleswaran | National COVID Cohort Collaborative (N3C) |
| Robert Hurley | National COVID Cohort Collaborative (N3C) |
| Robert T. Miller | National COVID Cohort Collaborative (N3C) |
| Saiju Pyarajan | National COVID Cohort Collaborative (N3C) |
| Sam G. Michael | National COVID Cohort Collaborative (N3C) |
| Samuel Bozzette | National COVID Cohort Collaborative (N3C) |
| Sandeep K. Mallipattu | National COVID Cohort Collaborative (N3C) |
| Satyanarayana Vedula | National COVID Cohort Collaborative (N3C) |
| Scott Chapman | National COVID Cohort Collaborative (N3C) |
| Shawn T. O’Neil | National COVID Cohort Collaborative (N3C) |
| Soko Setoguchi | National COVID Cohort Collaborative (N3C) |
| Stephanie S. Hong | National COVID Cohort Collaborative (N3C) |
| Steven G. Johnson | National COVID Cohort Collaborative (N3C) |
| Tellen D. Bennett | National COVID Cohort Collaborative (N3C) |
| Tiffany J. Callahan | National COVID Cohort Collaborative (N3C) |
| Umit Topaloglu | National COVID Cohort Collaborative (N3C) |
| Valery Gordon | National COVID Cohort Collaborative (N3C) |
| Vignesh Subbian | National COVID Cohort Collaborative (N3C) |
| Warren A. Kibbe | National COVID Cohort Collaborative (N3C) |
| Wenndy Hernandez | National COVID Cohort Collaborative (N3C) |
| Will Beasley | National COVID Cohort Collaborative (N3C) |
| Will Cooper | National COVID Cohort Collaborative (N3C) |
| William Hillegass | National COVID Cohort Collaborative (N3C) |
| Xiaohan Tanner Zhang | National COVID Cohort Collaborative (N3C) |
